# The *HOX* code of human adult fibroblasts reflects their ectomesenchymal or mesodermal origin

**DOI:** 10.1007/s00418-025-02362-9

**Published:** 2025-03-10

**Authors:** Lucie Pfeiferová, Michal Španko, Jana Šáchová, Miluše Hradilová, Kenneth J. Pienta, Jaroslav Valach, Vladimír Machoň, Barbora Výmolová, Aleksi Šedo, Petr Bušek, Pavol Szabo, Lukáš Lacina, Peter Gál, Michal Kolář, Karel Smetana

**Affiliations:** 1 Institute of Molecular Genetics, Czech Academy of Sciences, Prague, Czech Republic; 2 Faculty of Chemical Technology, Department of Informatics and Chemistry, University of Chemistry and Technology in Prague, Prague, Czech Republic; 3 First Faculty of Medicine, Institute of Anatomy, Charles University, Prague, Czech Republic; 4 First Faculty of Medicine and The General University Hospital, Department of Stomatology, Charles University, Prague, Czech Republic; 5 School of Medicine, Johns Hopkins University, James Buchanan Brady Urological Institute, Baltimore, USA; 6 First Faculty of Medicine, Institute of Biochemistry and Experimental Oncology, Charles University, Prague, Czech Republic; 7 First Faculty of Medicine, Charles University, BIOCEV, Vestec, Prague, Czech Republic; 8 First Faculty of Medicine and General University Hospital, Department of Dermatovenereology, Charles University, Prague, Czech Republic; 9 Faculty of Medicine, Department of Pharmacology, Pavol Jozef Šafárik University in Košice, Košice, Slovak Republic; 10 Department for Biomedical Research, East-Slovak Institute of Cardiovascular Diseases, Inc, Košice, Slovak Republic; 11 Faculty of Pharmacy, Department of Pharmacognosy and Botany, Comenius University in Bratislava, Bratislava, Slovak Republic; 12 Third Faculty of Medicine, Charles University, Prague Burn Center, Prague, Czech Republic

**Keywords:** Ectomesenchyme, Expression pattern, Fibroblasts, Homeobox genes, Mesoderm, Cancer-associated fibroblasts

## Abstract

Fibroblasts, the most abundant cell type in the human body, play crucial roles in biological processes such as inflammation and cancer progression. They originate from the mesoderm or neural-crest-derived ectomesenchyme. Ectomesenchyme-derived fibroblasts contribute to facial formation and do not express *HOX* genes during development. The expression and role of the *HOX* genes in adult fibroblasts is not known. We investigated whether the developmental pattern persists into adulthood and under pathological conditions, such as cancer. We collected adult fibroblasts of ectomesenchymal and mesodermal origins from distinct body parts. The isolated fibroblasts were characterised by immunocytochemistry, and their transcriptome was analysed by whole genome profiling. Significant differences were observed between normal fibroblasts from the face (ectomesenchyme) and upper limb (mesoderm), particularly in genes associated with limb development, including *HOX* genes, e.g., *HOXA9* and *HOXD9*. Notably, the pattern of *HOX* gene expression remained consistent postnatally, even in fibroblasts from pathological tissues, including inflammatory states and cancer-associated fibroblasts from primary and metastatic tumours. Therefore, the distinctive *HOX* gene expression pattern can serve as an indicator of the topological origin of fibroblasts. The influence of cell position and *HOX* gene expression in fibroblasts on disease progression warrants further investigation.

**Supplementary Information:**

The online version contains supplementary material available at 10.1007/s00418-025-02362-9.

## Introduction

Fibroblasts, vital architects of human tissues, play dynamic roles in both embryonic development and adult organ function through their structural support and intricate interactions with epithelial cells. Their roles extend to pathological processes, where they are pivotal in wound healing, fibrosis and/or inflammation. For example, fibroblasts are essential in wound repair, with dysfunction leading to chronic wounds or excessive scarring, such as hypertrophic or keloid scars (Čoma et al. 2021). They are also central to the progression of organ fibrosis and inflammatory diseases, including systemic sclerosis, liver and kidney fibrosis, rheumatoid arthritis and coronavirus disease 2019 (COVID-19)-related lung fibrosis (Gál et al. 2022; Deng et al. 2021; Kisseleva and Brenner 2021; Lomholt et al. 2023; Yuan et al. 2019).

In the context of cancer, fibroblasts within the tumour stroma, termed cancer-associated fibroblasts (CAFs), significantly influence tumour biology across various cancer types (Lacina et al. 2022; Plzák et al. 2010). Their activity appears broad and rather tumour-type unspecific (Dvořánková et al. 2012, 2005). Initially, CAFs were identified in biopsies via immunohistochemistry using anti-α-smooth muscle actin (SMA) antibodies, often conflated with myofibroblasts. However, not all stromal CAFs are myofibroblasts, and tumour variability in SMA-positive CAFs is significant (Novák et al. 2021). The lack of universally accepted CAF markers warrants further investigation.

Recent single-cell sequencing studies have underscored the functional diversity of fibroblasts in different tissues (Driskell and Watt 2015; Sriram et al. 2015; Vorstandlechner et al. 2020). This heterogeneity is partly attributable to their embryonic origins. Fibroblasts can originate from the mesoderm or the neuroectoderm via neural crest differentiation (Lynch and Watt 2018; Houzelstein et al. 2000; Le Lievre and Le Douarin 1975; LeBleu and Neilson 2020). In the head and neck region, for instance, fibroblasts can derive from both sources, with those in the facial region stemming from neural crest-derived ectomesenchyme. In contrast, those in the posterior region of the head originate from the mesoderm (Creuzet et al. 2005).

The *HOX* genes, a conserved family of transcription factors, are crucial for regulating craniocaudal development. Their expression is tightly controlled during embryogenesis (Deschamps and Duboule 2017). In the head and neck, the *HOX* gene expression is spatially patterned, with specific regions exhibiting characteristic expression profiles essential for normal development and function (Parker et al. 2018; Miyoshi et al. 2015; Živicová et al. 2017). Fibroblasts of the facial region, derived from neural crest-originated ectomesenchyme, are characteristically negative for *HOX* gene activity during development (Creuzet et al. 2005). Aberrant activity of *HOX* genes in typically *HOX*-negative regions, such as the first and second pharyngeal arches, correlates with facial developmental irregularities (Whiting 1997; Parker et al. 2018). In other body regions, such as the trunk and limbs, normal fibroblasts exhibit region-specific *HOX* gene activity both pre- and postnatally, affecting adult tissue functions (Miyoshi et al. 2015; Živicová et al. 2017; Hajirnis and Mishra 2021; Chang et al. 2002).

Despite extensive research, the stability of *HOX* gene activity in adult human fibroblasts of ectomesenchymal origin under various conditions remains poorly understood. Fibroblasts are an extremely heterogeneous cell type, influencing the tissue microenvironment in both normal and pathological states (Lynch and Watt 2018; Miki and Manresa 2023). However, this heterogeneity is often overlooked in research design. Therefore, we aimed to investigate whether the developmental *HOX* gene signature is preserved in adult fibroblasts under physiological and pathological conditions. Inspired by a previous study on CAF origin (Arina et al. 2016), we investigated the relationship between the origin of CAFs and *HOX* gene expression. Understanding whether CAFs originate locally or migrate to tumour sites from distant locations could provide valuable insights. By comparing transcriptome profiles of fibroblasts from the face (ectomesenchymal origin) and forearm (mesodermal origin), we sought to elucidate the differences in *HOX* gene expression postnatally. Our analysis extended to CAFs isolated from various tumours (including those from the face and other body parts, such as the brain). Gene expression of fibroblasts and its relation to their developmental origin may reveal the effects of postnatal age and pathological conditions (such as cancer) on the *HOX* gene profile.

## Material and methods

### Human subjects

Normal fibroblasts and fibroblasts from the pathological tissues were collected between 2017 and 2023, with the explicit informed consent of all involved donors, and after approval of the Ethics Committees of the following Prague hospitals: General University Hospital, University Hospital Královské Vinohrady, University Hospital in Motol, Na Homolce Hospital and Central Military University Hospital. We obtained residual tissue samples not needed for diagnostic purposes and used them for fibroblast isolation.

A collection of facial dermal fibroblasts (*n* = 6) was prepared from skin biopsies harvested from the facial skin of young adults (aged 20–30 years old). The position was standardised in all cases; the biopsy was taken in front of the external ear, approximately 2.5 cm ventral from the tragus. An identical number (*n* = 6, age-matched) of skin tissue samples was collected from the upper forearm skin (radial side, approximately 7.5 cm distal from the flexural line). The standardised biopsy sites are presented in the schematic figure (Supplementary Fig. 1). Other analysed fibroblasts were collected from various body parts, including the oral cavity and internal organs, such as the pancreas (Table 1). The pathological samples were selected to cover various CAFs and skin samples with abnormal immune responses, e.g., systemic sclerosis (SSF). SSF is known to exhibit unique pathological behaviour, including excessive activation and resistance to apoptosis, which are features also seen in CAFs. Subgalear fibroblast were selected as the best available control for the intracranial CAFs. For ethical reasons, we could not collect cells other than these cells excised during the approach to the brain tumours. Samples originating from regions derived from neuroectoderm (face, oral cavity, forebrain) were considered as ectomesenchymal. Intracranial cells are further described in Supplementary Table 1.

**Table 1:** Source of the fibroblasts according to diagnosis and location

Source	Abbreviation	Number of samples	Number of mesoderm samples	Number of ectomesenchyme samples
Normal dermal fibroblasts – face^*^	Face	6	0	6
Normal dermal fibroblasts – forearm/trunk/leg^*^	Forearm/trunk/leg	6/6/6	6/6/6	0
Normal dermal fibroblasts	DF	56	25	31
Normal dermal fibroblasts − deep dermis and adipose body of hypodermis	AF	4	4	0
Normal fibroblasts from oral mucosa	MuF	8	0	8
Normal fibroblasts from the soft tissue between galea aponeurotica and calvary periost	Subgalear	5	5	0
Fibroblasts (dermal) from systemic sclerosis	SSF	6	6	0
Fibroblasts from epileptogenic focus	PRE	3	0	3
Fibroblasts from the pancreas − collected distant from ductal adenocarcinoma tissue	PANF_control	2	2	0
CAFs from basal cell carcinoma	BCCF	23	3	20
CAFs from squamous cell carcinoma	SCCF	13	3	10
CAFs from sporadic keratoacanthoma	KAF_S	7	7	0
CAFs from BRAF inhibitor-induced keratoacanthoma	KAF_I	13	13	0
CAFs from malignant cutaneous melanoma	MELF	6	6	0
CAFs from ductal adenocarcinoma of the pancreas	PANF	8	8	0
CAFs from glioblastoma	GBM	6	0	6
CAFs from breast cancer brain metastasis	META	3	Unknown origin	Unknown origin
CAFs from lung cancer brain metastasis	META	4	Unknown origin	Unknown origin
CAFs from spindle cell poorly differentiated sarcoma brain metastasis	META	1	Unknown origin	Unknown origin
CAFs from primary serous peritoneal carcinoma brain metastasis	META	1	Unknown origin	Unknown origin
CAFs from a brain metastasis of clear cell renal carcinoma	META	1	Unknown origin	Unknown origin

Normal dermal fibroblasts from the trunk and leg were analysed by immunocytochemistry only

^*^Samples with exactly defined location

The total number of samples derived from normal and pathological tissues was 85 and 97, respectively. Transcriptome profiling of these samples was performed using either microarrays (70 normal samples and 76 pathological samples) or RNA sequencing (RNA-Seq) (17 normal samples and 19 pathological samples). The technology used for sample profiling is specified in the figure legends.

### Fibroblast isolation and characterisation

Fibroblasts from normal and pathological tissues were isolated and characterised as described earlier (Dvořánková et al. 2019). In the case of glioblastomas, brain metastases, pharmacoresistant epilepsy and subgalear fibroblasts, fibroblasts were isolated by direct magnetic-activated cell sorting (MACS) using a fibroblast-specific kit (Fibroblast MicroBeads, Miltenyi, Bergisch Gladbach, Germany) according to manufacturer’s instructions. The purity of the cells was evaluated using a panel of antibodies (Supplementary Table 2), as described previously (Balaziova et al. 2021). Fibroblasts were expanded in Dulbecco’s modified Eagle’s medium [DMEM with high glucose content (4.5 g/L)], supplemented with 10% foetal bovine serum (both from Biosera, Nuaille, France) with antibiotics (penicillin 100 IU/mL, streptomycin 100 µg/mL and gentamycin 100 µg/mL, all Sigma Aldrich, Prague, Czech Republic), and maintained in 5% CO_2_ atmosphere and 37 °C in a humidified incubator. Fibroblasts from early passages (before passage no. 5) were used to measure the cell volume, growth characteristics and transcriptomic analyses.

For cell counting and analyses, cells were routinely harvested in trypsin (0.25%) and ethylenediaminetetraacetic acid (EDTA) (0.02%) solution (Biosera, Nuaille, France) and vigorously resuspended in the culture medium. For cell counting, the final cell suspension (200 μL) was diluted 1:50 using Isoton II diluent (Life Sciences, Indianapolis, USA) and measured using a Beckman Coulter Particle Counter Z2 (Life Sciences, Indianapolis, USA) following the manufacturer’s protocol. The cells were counted between the lower and upper thresholds of 12 and 24 μm, respectively. The size distribution measurement of the cell population within this range was plotted in 256 identical bins and statistically evaluated using a one-way ANOVA test (using GraphPad Prism software, version 8.0.1).

For the proliferation assay, 5000 cells were seeded in a 96-well plate, and the confluence was monitored using an IncuCyte S3 live-cell analysis instrument (Sartorius, Goettingen, Germany) every 2 h for 6 days. The normalised data curve (normalisation to initial confluence value) was plotted using GraphPad Prism. Two biological replicates and six technical replicates were used for each group.

For immunocytochemical analysis, we cultured cells from 12 donors (6 donors of facial fibroblasts and 6 donors of matched forearm, leg and trunk fibroblasts, approximately 20,000 cells/cm^2^) on sterile microscopic slides for 48 h. The cells were fixed in buffered paraformaldehyde (2%) solution (Sigma–Aldrich, Prague, Czech Republic) and subsequently permeabilized by Tris-buffered saline (TBS) with 0.2% Tween 20 (Sigma–Aldrich, Prague, Czech Republic). Endogenous peroxidase was blocked by incubation with 3% hydrogen peroxide in TBS at room temperature for 20 min. To block non-specific protein binding and dilute primary antibodies, we used the Universal IHC Blocking/Diluent (Leica, Wetzlar, Germany). The antibodies in 1:100 dilution were used for fibroblast characterisation (Supplementary Table 2).

After overnight incubation at 4 °C, the slides were washed, and the immunohistochemical reaction was developed using Histofine^®^ High Stain™ HRP (MULTI) and N-Histofine^®^ Simple Stain™ AEC Solution (both Nichirei Biosciences Inc, Tokyo, Japan). Slides were counterstained in Gill´s haematoxylin and mounted in Biomount Aqua (both Baria, Prague, Czech Rep.). Negative controls were performed using species-specific isotype control antibodies (Thermo Fisher Scientific, Waltham, MA, USA). The bright-field images were acquired using a Leica DM2000 microscope equipped with LASx software.

### Transcriptome profiling by microarrays

Total RNA was isolated using the RNeasy Micro Kit (Qiagen, Hilden, Germany) according to the manufacturer’s protocol. The quantity and quality of RNA were analysed using Agilent 2100 Bioanalyzer (Agilent Technologies, Santa Clara, CA, USA). All RNA samples had RNA integrity number (RIN) above 9. Total RNA (200 ng) was amplified using the Illumina TotalPrep RNA amplification kit (Ambion; Thermo Fisher Scientific, Waltham, MA, USA), and 750 ng of the amplified RNA was hybridised on Illumina HumanHT–12 v4 chips (Illumina, San Diego, CA, USA) following the manufacturer’s protocol.

Raw data were processed using the oligo (Carvalho and Irizarry 2010) and limma (Ritchie et al. 2015) packages of R/Bioconductor. Data were background corrected using the normal–exponential model and quantile normalised. Batch effects were corrected using the sva (Leek et al. 2012) package. Log_2_-transformed normalised expression data were used for heatmap visualisation using the ComplexHeatmap (Gu et al. 2016) R package.

### Transcriptome profiling by RNA-Seq

Total RNA was prepared from tissue cultures by the RNeasy Micro Kit (Qiagen, Hilden, Germany). RNA quality was controlled by Agilent 2100 Bioanalyzer, and only samples with RIN above 7 were used for further preparations. Sequencing libraries were prepared from a 1 μg input amount of total RNA by a KAPA mRNA HyperPrep Kit, including polyA selection and barcoding with a KAPA UDI Adapter Kit (all by Roche). An equimolar pool of libraries was sequenced by the Illumina NextSeq 500 platform using 75 nt long single-end reads.

Technical quality control and gene quantification were done using the nf-core/rnaseq v3.4 bioinformatics pipeline (Ewels et al. 2020) with STAR mapping (Kim et al. 2015) and Salmon quantification (Patro et al. 2017). GRCh38 (ensEMBL assembly version 104) was selected as the reference genome (Howe et al. 2021). Genes expressed only in a single sample were discarded. The DESeq2 (v1.38.3) (Love et al. 2014) Bioconductor (v3.16) R package was used to identify differentially expressed genes. Significant changes in gene expression were defined by a two-fold change in gene expression intensity and false discovery rate (FDR) < 0.1. Shrunken log-fold change estimates were used [adaptive shrinkage estimator (Stephens 2017)]. The gene set enrichment analysis (Subramanian et al. 2005) was performed on the Gene Ontology terms (Ashburner et al. 2000; Aleksander et al. 2023) using the ClusterProfiler (Wu et al. 2021) package. Boxplots present log_2_-transformed quantile normalised limma (Ritchie et al. 2015) TPM values from Salmon. The boxes display median, upper and lower quartiles, whiskers denote range of values with outliers excluded. Heatmaps were created using the ComplexHeatmap (Gu et al. 2016) package on standardised quantile-normalised TPM values (*z*-score).

The transcriptomic data sets used in this article are available in the ArrayExpress database (https://www.ebi.ac.uk/biostudies/arrayexpress) under the accession numbers E-MTAB-13241, E-MTAB-13242 and E-MTAB-13243. Any additional information required to reanalyse the data reported in this paper is available from the corresponding authors upon reasonable request.

## Results

### Comparison of facial fibroblasts of ectomesenchymal origin and forearm fibroblasts of mesodermal origin

**Fig. 1:**
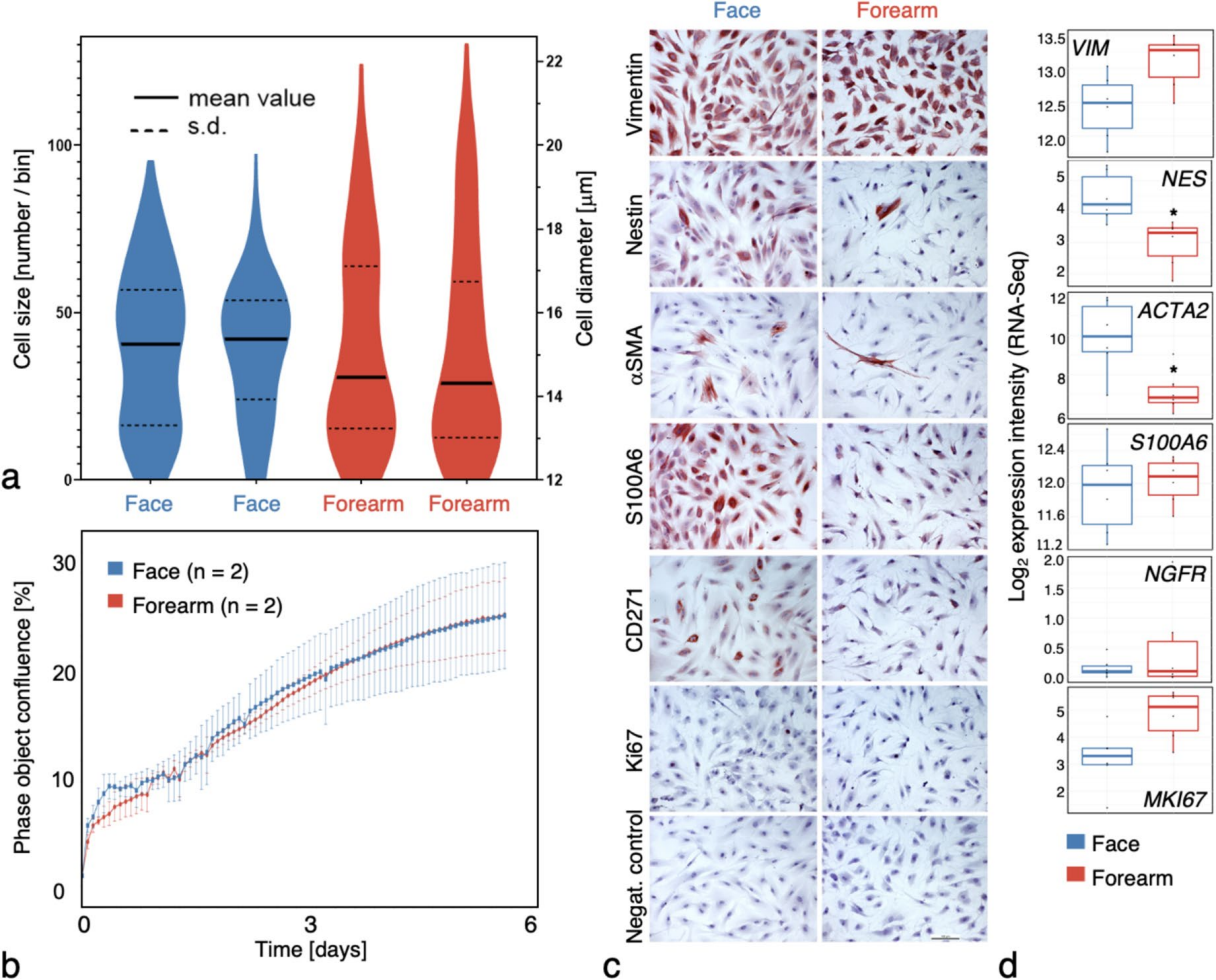
Measurements of cell volume (**A**) and growth characteristics (**B**) along with immunocytochemical detection of vimentin, nestin, αSMA, CD271, S100, and Ki67 (**C**) in adult dermal fibroblasts derived from the face and forearm. Expression of the transcripts for all studied proteins is also shown (**D**). Facial fibroblasts were somewhat larger than cells harvested from the dermis of the forearm (**A**), but their growth kinetic was identical (**B**). [Growth curves were plotted as normalised to the baseline scan confluence value. Error bars represent standard deviation (s.d.) calculated from the well replicates (*n* = 6 technical replicates) for each fibroblast type (face *n* = 2, forearm *n* = 2 biological replicates); the observed differences were not statistically significant.] (**C**) The expression of vimentin was not influenced by fibroblast origin. The expression of nestin and αSMA was significantly higher (^*^p < 0.05) at both mRNA and protein levels in facial fibroblasts. Although the positivity for S100A6 protein was higher in facial fibroblasts, the transcript level (*S100A6*) was the same in both fibroblast types. CD271 was higher in facial fibroblasts than in fibroblasts from the forearm at the protein level, but there was no difference at the mRNA level. The type of fibroblasts did not influence the expression of Ki67. Negative control exhibited no positivity. The scale bar indicates 200 μm

Both types of dermal fibroblasts were large spindle-shaped cells usually possessing several processes. Their morphology varied according to the population density. However, no statistically significant difference in morphology was observed across the compared populations. Facial fibroblasts were slightly larger; however, the difference was not statistically significant (cell diameter, *p*-value = 0.19) (Fig. 1A). The growth kinetics of facial- and limb-originated fibroblasts were identical (Fig. 1B). According to the immunocytochemical analysis, the expression of vimentin was approximately the same in both types of fibroblasts (Fig. 1C). Conversely, immunocytochemical detection demonstrated that the expression of nestin, α-smooth muscle actin (αSMA), S100 protein and CD271 (NGFR) was higher in facial- than in forearm-originated fibroblasts (Fig. 1C). The expression of proliferation marker Ki-67 was similar in both types of fibroblasts. The higher expression of *nestin* and *αSMA* was also confirmed at the mRNA level (Fig. 1D).

**Fig. 2:**
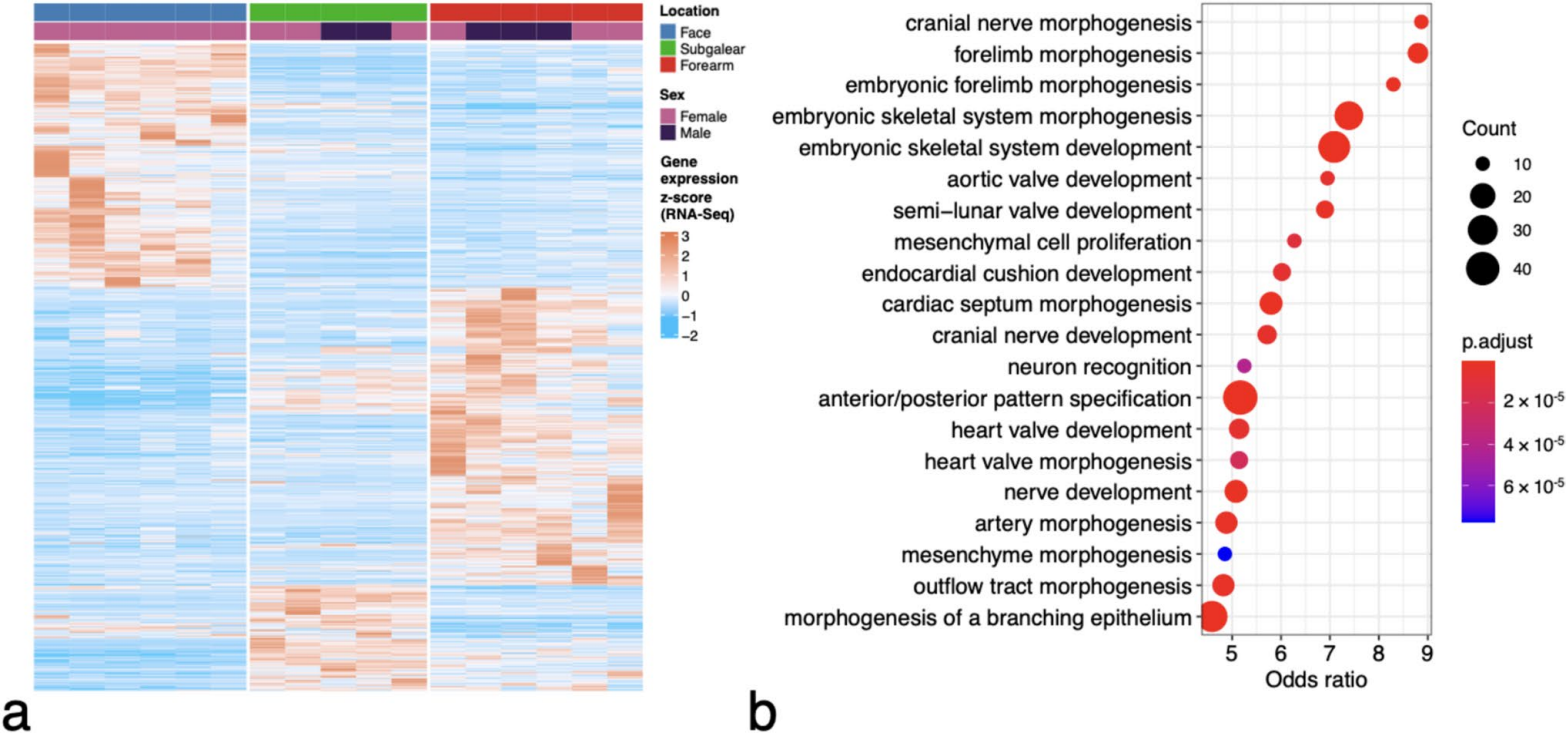
Heatmap demonstrating the difference between the expression profiles of dermal fibroblasts prepared from the forearm and face (**A**). Subgalear fibroblasts, presumably also mesoderm-originated, are included for comparison. Differences between adult ectomesenchyme-originated fibroblasts from the face and mesoderm-originated fibroblasts from the forearm reflect the regulatory cascades important for morphogenesis and development (**B**)

**Fig. 3:**
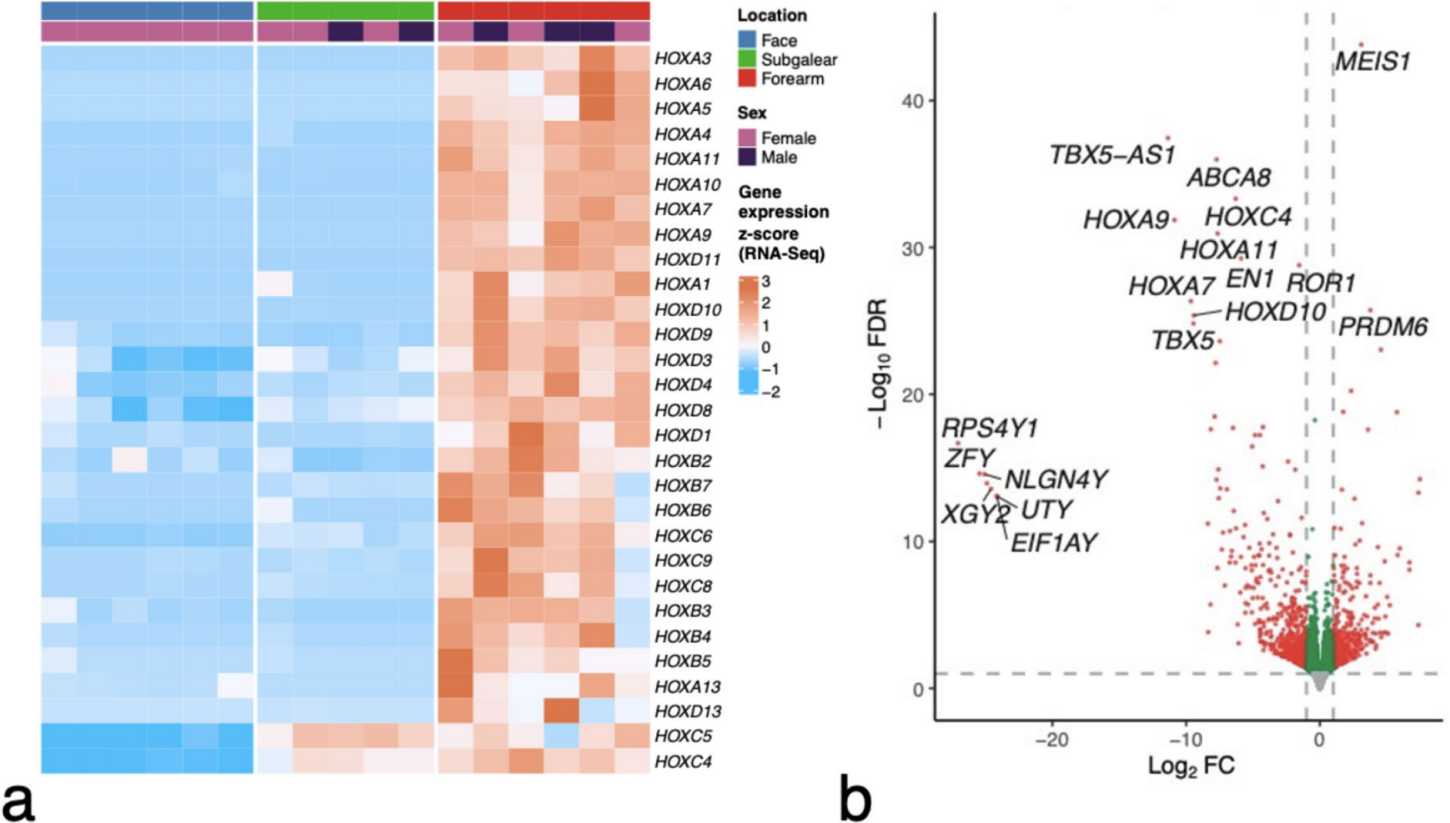
Heatmap of homeobox genes that are differentially expressed between dermal fibroblasts prepared from the human face (ectomesenchyme) and the forearm (mesoderm) (**A**). Subgalear fibroblasts are included for comparison. The volcano plot (**B**) demonstrates that the homeobox genes are the most upregulated genes in fibroblasts prepared from the forearm. This difference is primarily attributable to the absence of activity of *HOX* genes in facial fibroblasts. Since the facial fibroblasts were collected only from female donors, the volcano plot also shows a distinct set of Y-linked genes. The *HOX* gene signal, however, does not depend on the donor’s sex

RNA-Seq transcriptome analysis revealed significant differences in the transcription profile between ectomesenchyme-originated fibroblasts prepared from the face. and mesoderm-originated fibroblasts from the upper forearm of adult donors, where 959 genes were differentially expressed (Fig. 2A). Gene set enrichment analysis showed that Gene Ontology terms related to development and morphogenesis were the most enriched in differentially expressed genes (Fig. 2B). The expression of *HOX* genes was well marked in fibroblasts prepared from the forearm in comparison with fibroblasts from the face, as shown in the volcano plot and heatmap in Fig. 3. Conversely, the expression of *MEIS1* and *PRDM6* genes was upregulated in facial fibroblasts (Supplementary Fig. 2).

**Fig. 4:**
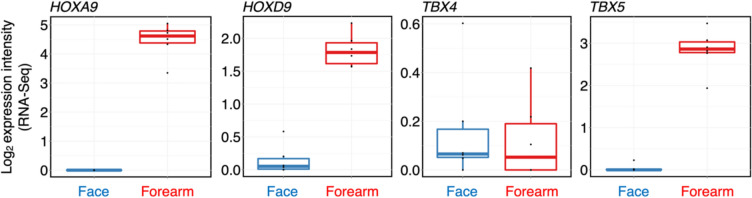
Examples of the *HOX* genes participating in developing vertebrate limbs and the *TBX5* gene controlling the formation of upper limbs. The genes displayed significantly stronger expression in fibroblasts prepared from the forearm than in fibroblasts prepared from facial skin. A negligible activity of *TBX4*, which is known to be important in the development of the lower limb, was observed in both facial- and forearm-originated fibroblasts

When we focused our interest on genes participating in the development of the upper and lower limbs, we detected significantly higher activity of the *HOXA9*, *HOXD9*, *HOXA10*, *HOXD10*, *HOXA11*, *HOXD11*, *HOXA13* and *TBX5* genes in the forearm fibroblasts (all FDR < 0.001). As expected, the activity of the *TBX4* gene, which participates in lower limb development, was not upregulated (FDR > 0.9, Fig. 4; Supplementary Fig. 3).

To test the applicability of this gene expression pattern for identifying the origin of fibroblasts, we employed a collection of fibroblasts isolated from subgalear soft connective tissue. These samples (isolated from occipital to parietal regions) provided cells that exhibited the activity of *HOXC5* and *HOXC4* genes, as demonstrated in the heatmap (Fig. 3). Notably, this activity of *HOX* genes strikingly differed from the dermal fibroblasts isolated from the viscerocranium and forearm fibroblasts, as depicted in Figs. 2 and 3.

### Verification of selected HOX proteins by immunocytochemistry

**Fig. 5:**
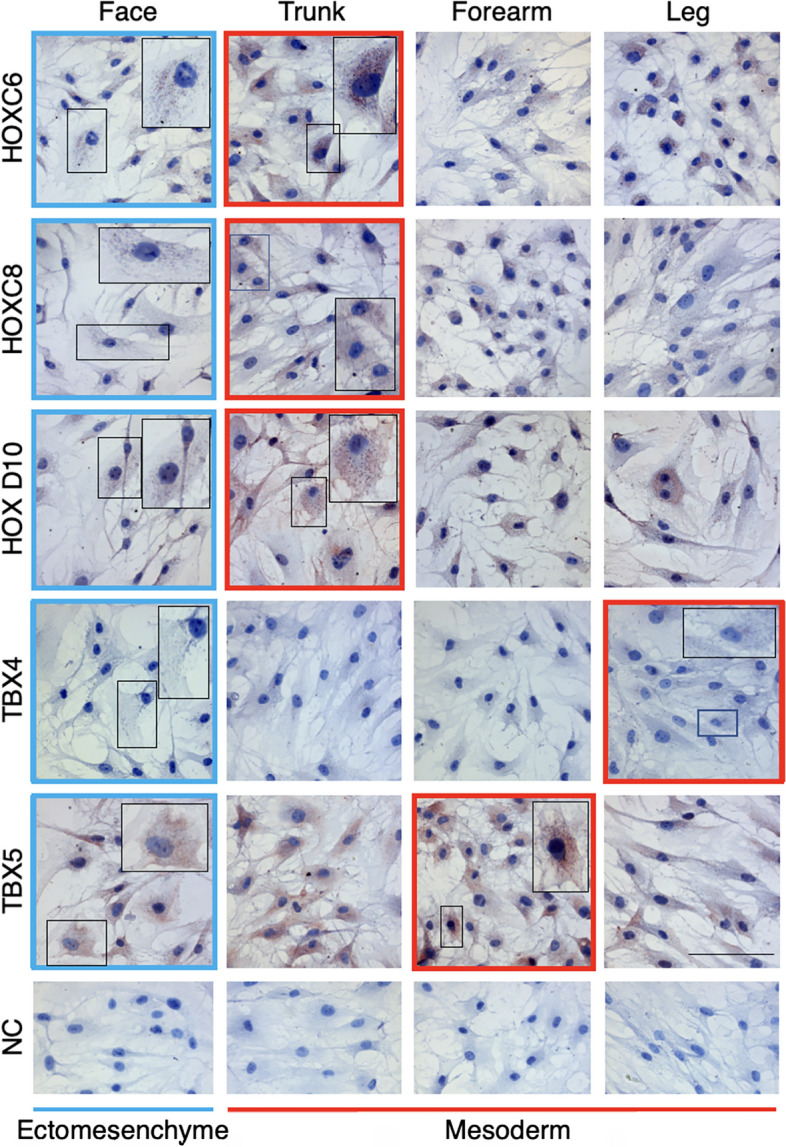
Immunocytochemical detection of HOXC6, HOXC8, HOXD10, TBX4 and TBX5 in normal dermal fibroblasts prepared from the face (ectomesenchyme), trunk, forearm and leg (all mesoderm). Nuclear positivity was detected in none of the fibroblast types. The lowest cytoplasmic granular signal was present in facial cells (blue frames), and the strongest HOXC6, HOXC8 and HOXD10 signals in the cells prepared from the trunk (red frames). The positivity for TBX4 was extremely low, yet the cells from the lower limb (red frame) displayed a clear signal. The highest signal for TBX5 was observed in fibroblasts from the forearm (red frame). A characteristic cell from the marked position of the panels with the lowest and strongest signals is detailed in the insets. Negative control (NC) was included to show the specificity of the reaction. The scale bar represents 200 μm

Selected proteins, i.e., HOXC6, HOXC8, HOXD10, TBX4 and TBX5 were also detected by immunocytochemistry in cultured fibroblasts originating from ectomesenchyme (face) and mesoderm (trunk, forearm and leg). We observed no signal of the presence of these proteins in the cell nucleus. The specific positivity of HOX proteins, in the form of granules, was detected in the cytoplasm of all studied types of fibroblasts. It was very low in facial cells (ectomesenchyme) and the strongest in the trunk (Fig. 5). Concerning the presence of products of *TBX4* and *TBX5* genes, the very low signal for TBX4 protein was observed only in the cytoplasm of leg-originated fibroblasts, and the TBX5 protein signal was stronger in fibroblasts from the forearm than in the cells from the leg (Fig. 5), reflecting the important role of these proteins in the development of the lower and upper limbs, respectively.

### Fibroblasts from different pathological tissues retain the expression activity of the homeobox genes

**Fig. 6:**
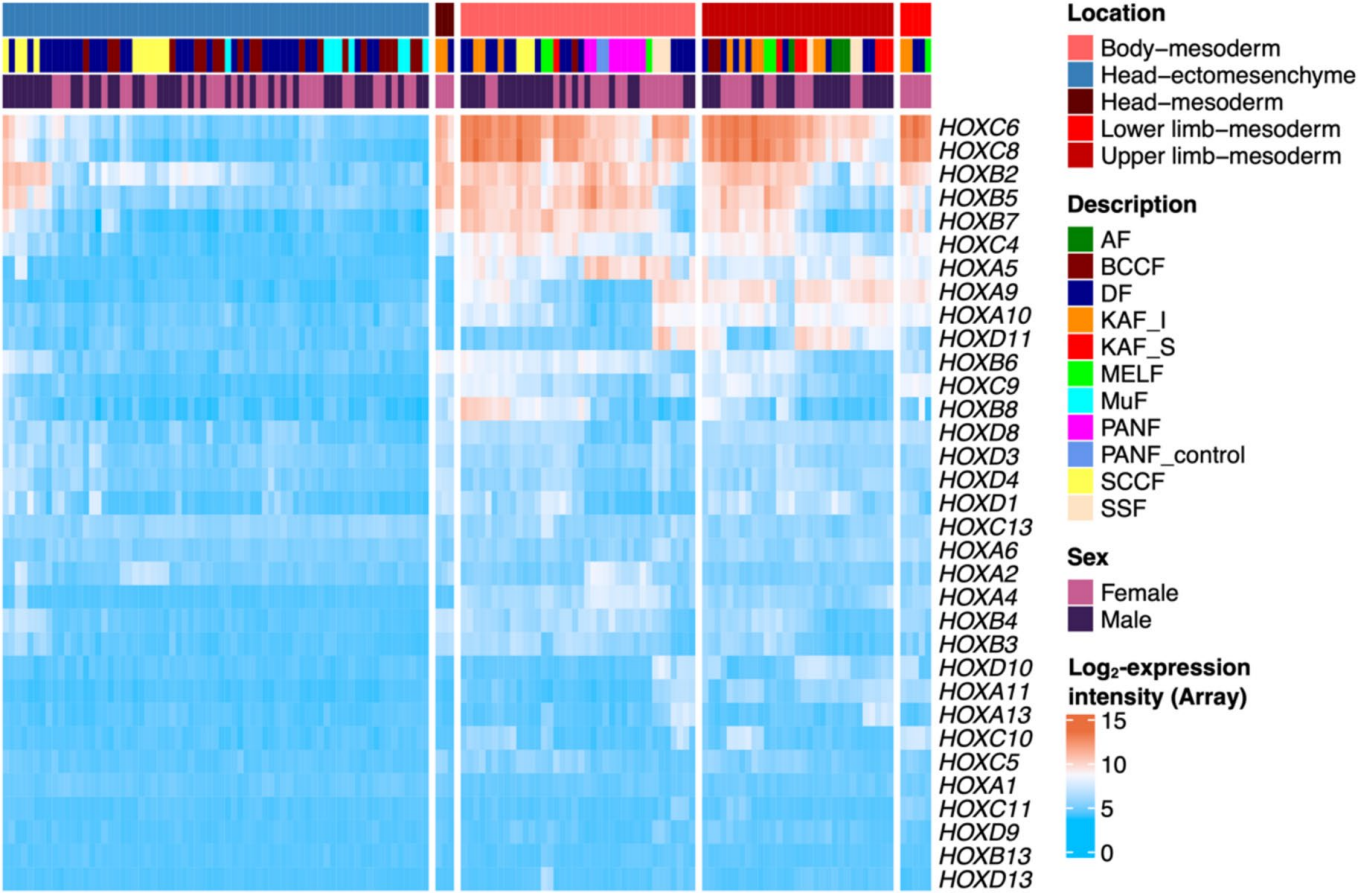
The expression profile of *HOX* genes in fibroblasts prepared from different pathological tissues, including CAFs, respects the mesodermal or ectomesenchymal origin of the cells. See Table 1 for abbreviations

The *HOX* gene expression pattern was evaluated in a broad collection of CAFs isolated from various primary or secondary tumours and several other pathological tissues (summarised in Table 1). When the source of tissue was in the body areas where fibroblasts are of mesodermal origin, fibroblasts prepared from these pathological tissues, including CAFs, demonstrated high expression of the *HOX* genes. We also analysed a sample of activated fibroblasts originating from mesoderm prepared from a patient suffering from systemic sclerosis. Similarly to mesoderm-originated normal dermal fibroblasts and activated CAFs, these SSF cells expressed *HOX* genes (Fig. 6).

**Fig. 7:**
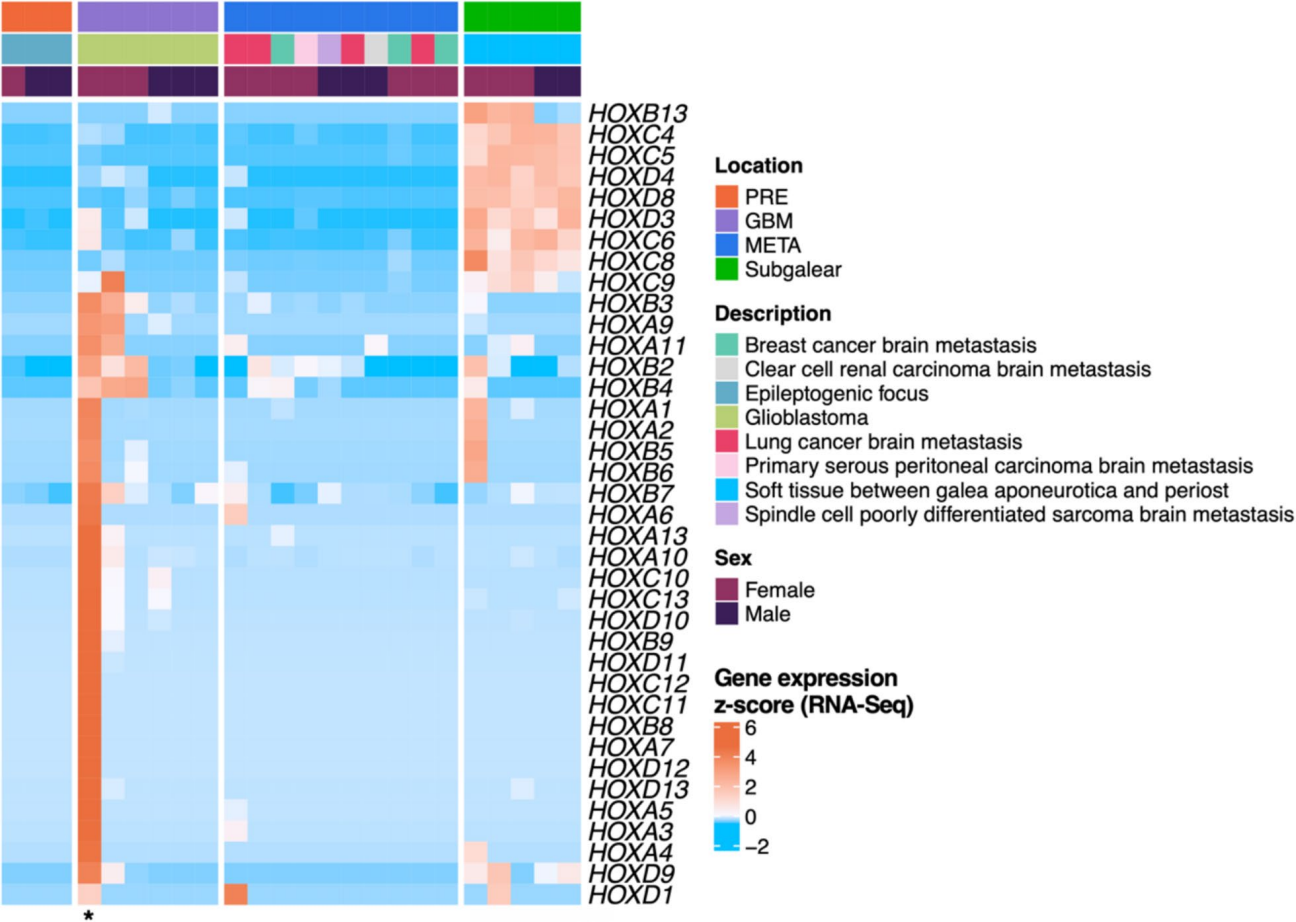
CAFs prepared from human glioblastoma (GBM) samples and brain metastases of malignant tumours (META) exhibited negligible activity of the *HOX* genes. Similarly, fibroblasts from the epileptogenic foci of the brain (PRE) exhibit no activity of *HOX* genes, in contrast to fibroblasts prepared from the subgalear soft tissue covering the dorsal part of the skull. The sample NCH353G, denoted by an asterisk, was later rediagnosed and may contain tumour-originated mesenchymal cells

Conversely, CAFs from tumours arising in the ectomesenchyme-dependent areas were devoid of *HOX* gene expression, with scarce exceptions (Fig. 6). A similar trend was confirmed in mesenchymal cells isolated from human glioblastomas (GBM, malignant primary brain malignant tumour) and secondary brain tumours (metastases of various cancer types to the brain) (Fig. 7). Of note, fibroblasts prepared from epileptogenic foci in the brain were also devoid of *HOX* gene activity (Fig. 7).

### Determination of the effect of sex on homeobox gene expression

Next, we decided to determine whether the homeobox code depends on the sex of the patients by comparing gene expression in female and male samples within individual sample groups. Only the groups, where each sex was represented by at least three samples were selected for the analysis (DF from head, DF from body, forearm, BCCF, PANF, META, and GBM). KAF_I samples were disregarded owing to possible influence of the biological treatment. The observed significantly differentially expressed homeobox genes are presented in Supplementary Table 3. In the normal cells, we did not observe any reproducible differences. In the GBM group, we noticed changes in several homeobox genes, e.g., *NKX3-1*, *HOXA10*, *PROX1*, *EN2*, *DLX1* and *HOXB3* (all FDR < 0.05 and at least fourfold deregulation), which were all upregulated in the samples from female patients. In PANF, we observed the strongest changes in expression of the *IRX4* and *NKX2-5* genes. As the differences were tissue specific, we could not prove any general difference in the homeobox gene expression with respect to patient sex.

## Discussion

Our study revealed that adult facial dermal fibroblasts, originating from the ectomesenchyme, were morphologically very similar to dermal fibroblasts of mesodermal origin from the forearm. However, the cells differed in expression of homeobox genes. Consistent with their origin, adult facial fibroblasts exhibited negligible *HOX* gene activity. Conversely, *HOX* genes were expressed in all adult fibroblasts of mesodermal origin (Hahn et al. 2021; Miyoshi et al. 2015). Notably, genes such as *HOXA/D9*, *HOXA/D10*, *HOXA/D11* and *HOXA13*, which participate in limb primordium development, were significantly upregulated in dermal fibroblasts from the adult forearm. These results are supported by murine developmental models (Desanlis et al.^2020^) and studies of *HOX* gene activity in adult mouse limb fibroblasts (Okubo et al. 2018). The *TBX4* and *TBX5* genes, which determine upper and lower limb discrimination (Duboc and Logan 2011; Duboc et al. 2021), also showed distinct patterns, with *TBX5* highly active in forearm fibroblasts, reflecting their positional memory.

Analysis of the adult dermal fibroblasts from the ectomesenchyme and mesoderm exhibited well-conserved transcriptomic programs associated with development, including forelimb and occipitotemporal region morphogenesis (Supplementary Fig. 2). Facial fibroblasts of ectomesenchymal origin expressed *MEIS1* and *PRDM6* more actively than mesodermal-origin fibroblasts. *MEIS1*’s roles include neural crest development, regulation of cell proliferation, stemness and differentiation (Aksoz et al. 2017; Maeda et al. 2001; Jiang et al. 2021; Blasi and Bruckmann 2021). The function of the *PRDM6* gene is associated with neural crest cell function and heart development (Hong et al. 2022). The detection of these genes in facial fibroblasts reflects their developmental origin and supports the idea that *HOX* gene expression correlates with location. Using a consistent control, collected from healthy donors, we explored the potential role of the positional *HOX*-coded signature in various pathologies. Our data confirm the conservation of the *HOX* code in normal and cancer-associated fibroblasts (CAFs) isolated from a broad panel of tissues and cancer types in adults, including skin, oral mucosa, skin cancers (basal cell carcinoma, squamous cell carcinoma, melanoma), non-tumorous pancreatic tissue from patients with pancreatic cancer, pancreatic ductal adenocarcinoma, brain tissue and malignant brain tumours (glioblastoma and brain metastases).

The origin of CAFs has been a long-standing topic in cancer biology (Orimo and Weinberg 2006). Suggested source populations include resident fibroblasts, myofibroblasts, pericytes, preadipocytes, smooth muscle cells, mesenchymal stem cells (MSCs), and bone marrow-derived progenitor cells (BM-MSCs) (Karnoub 2007; Li et al. 2021). A broad comparison of BM-MSCs from different body parts revealed that most BM-MSCs express *HOX* genes, with specific sets varying by anatomical origin (Picchi et al. 2013). These *HOX* codes, characteristic of MSCs, are maintained during differentiation, indicating an intrinsic property (Ackema and Charité, 2008). Clinically relevant BM-MSCs typically show increased expression of *HOXA9*, *HOXA10*, *HOXB4*, *HOXB7*, *HOXC8*, *HOXC10* and *HOXD8* (Coenen et al. 2015). The positional memory of MSCs is evidenced by the maintenance of *HOX* code expression in culture (Wagner et al. 2006). In our dataset (Fig. 6), the listed *HOX* genes were not highly expressed in CAFs from intracranial metastases, suggesting that BM-MSCs are unlikely to be the primary source of these stromal cells. However, this does not exclude their regulatory role in brain metastases. From this point of view, our data generally suggest that local cells predominantly serve as the source of CAFs in almost all samples evaluated, with only a few exceptions to this principle.

One particularly intriguing group worthy of closer attention is fibroblasts isolated from glioblastoma samples. In several CAF samples derived from these highly malignant brain tumours, we observed a variable number of *HOX* genes expressed at varying intensities (Fig. 7). This was in stark contrast to control samples from pharmacoresistant epilepsy foci, where *HOX* gene transcription was generally silent, as expected, owing to their ectomesenchymal origin. Interestingly, subgalear fibroblasts from very proximal positions on the outer side of cranial bones expressed a panel of *HOX* genes with prominent intensity, aligning with the expected mesodermal development of the parieto-occipital region (Carlson 2018). Notably, the *HOX* gene transcriptional profile in GBM CAFs was mostly non-overlapping with subgalear fibroblasts, suggesting a distant origin of CAFs in GBM, possibly including mesenchymal stem cells or circulating fibrocytes of mesodermal origin (Lacina et al. 2022; Busek et al. 2016). Another potential source of CAFs in GBM is vascular wall-derived MSCs (VW-MSCs), located within the vascular stem cell niche or vasculogenic zone of the blood vessel wall. These MSCs are characterized by increased expression levels of *HOXB7*, *HOXC6*, *HOXC8* and several other *HOX* genes (Klein et al. 2013). In one case, the glioblastoma was later rediagnosed as gliosarcoma, and thus, the *HOX* gene expression may stem from contamination of CAFs by tumour mesenchymal cells. Our findings suggest that, at least in some patients, distant migrating populations can be a potential source of CAFs in GBM. This further highlights the *HOX* code as a master regulator of cellular identity (Klein 2021).

Linking cancer with long-standing inflammatory conditions presents numerous clinically relevant aspects (Lacina et al. 2019). The regulators of immunity and inflammation, such as epigenetic modifications (Rath et al. 2022), may lead to aberrant promoter methylation of various genes (Jurdziński et al. 2020) and dysregulation of their expression activity. To explore whether such dysregulation occurs in the *HOX* genes, dermal fibroblasts from patients suffering from systemic sclerosis (SSF) were included in our analysis. Data on fibroblasts are limited, but in the context of CAF biology, activated synovial fibroblasts from patients with autoimmune joint damage produce factors according to joint position reflected in their *HOX* code (Frank-Bertoncelj et al. 2017). These factors influence the clinical status of arthritis. In this context, robust data suggest that *HOX* gene expression or dysregulation influences cancer cell properties, with clear clinical relevance (Belpaire et al. 2022; Morgan et al. 2022; Wang et al. 2022). Both cancer-inhibiting and cancer-supporting roles have been reported. It remains unclear how the absence or expression of the *HOX* genes might affect cancer progression, as observed in leukaemia and solid tumours (Awgulewitsch 2003; Feng et al. 2021; Xu et al. 2022). Despite extensive data on *HOX* gene expression in cancer cells, information on their expression in CAFs, and their disease significance, remains limited (Wang et al. 2020).

Our study provides valuable insights into the *HOX* signature in fibroblasts of different embryonic origins but has certain limitations and characteristics of a pilot study. The analysis was conducted on fibroblasts isolated from normal skin and tumours and from patients with systemic sclerosis, cultured from the second to fifth passage. While the uniformity of results supports the conclusions, the artificial conditions of in vitro cell culture present some limitations. We quantified *HOX* gene expression in three primary cell lines of BCCF and melanoma. The results of this limited experiment suggested that the expression does not depend on cultivation time (4–24 weeks) or on cultivation conditions (Petri dish versus xenografts) (data not shown). Although our study includes a relatively large collection of solid tumours with uniform results, the number of samples for each diagnosis is relatively small, limiting the generalizability of our findings. Gene expression was verified in only a few representative samples at the protein level. This highlights the necessity for future studies to include comprehensive protein validation. In addition, single-cell sequencing would be essential to determine the heterogeneity of CAFs within individual tumours, allowing for a comparison of *HOX* gene expression with the activity of other genes. We did not detect any general changes in homeobox gene expression between sexes. However, we observed changes in their expression between female and male patients suffering from glioblastoma or pancreatic carcinoma. These topics will represent the next steps in our research.

From a practical standpoint, our data highlight the critical importance of considering the embryonic origins of fibroblasts in biomedical research. The specificity of the ectomesenchyme-based cancer microenvironment, often overlooked, is crucial for accurate experimental outcomes. Proper fibroblast controls are essential and mixing fibroblasts of different origins (ectomesenchyme versus mesoderm) is incorrect and can lead to misleading results. Neglecting the developmental origin of fibroblasts can lead to misinterpreted data, even with advanced ‘omics’ methods. Considering their origin is crucial for the validity and applicability of research findings in cancer biology and regenerative medicine.

## Supplementary Information

Below is the link to the electronic supplementary material.

Supplementary file1 (DOCX 248 KB)

## Data Availability

Transcriptomic data have been submitted to the ArrayExpress database with the following accession numbers: https://www.ebi.ac.uk/biostudies/arrayexpress/studies/E-MTAB-13241. https://www.ebi.ac.uk/biostudies/arrayexpress/studies/E-MTAB-13242. https://www.ebi.ac.uk/biostudies/arrayexpress/studies/E-MTAB-13243.
