# Supplementary material for: The HOX code of human adult fibroblasts reflects their ectomesenchymal or mesodermal origin
Source: Histochem Cell Biol. 2025 Mar 10;163(1):38. doi: 10.1007/s00418-025-02362-9 (PMC11893657; doi:10.1007/s00418-025-02362-9)
Supplement: Supplementary file 1 — Supplementary file1 (DOCX 248 KB) [file 418_2025_2362_MOESM1_ESM.docx]

# The *HOX* code of human adult fibroblasts reflects their ectomesenchymal or mesodermal origin

*Histochemistry and Cell Biology*

Lucie Pfeiferová^1,2*^, Michal Španko^3,4*^, Jana Šáchová^1^, Miluše Hradilová^1^, Kenneth J. Pienta^5^, Jaroslav Valach^4^, Vladimír Machoň^4^; Barbora Výmolová^6^, Aleksi Šedo^6^, Petr Bušek^6^, Pavol Szabo^3^, Lukáš Lacina^3,7,8^, Peter Gál^9,10,11,12^, Michal Kolář^1,2^, Karel Smetana Jr.^3,7^

**Suppl. Tab. 1**: Diagnosis of brain tissue samples used for the derivation of fibroblast cultures and their original localization in the brain.

| **Source** | **Sample** | **Diagnosis** | **Tumour localization** |
| --- | --- | --- | --- |
| Fibroblasts from epileptogenic focus | NCH236G | Pharmacoresistant epilepsy | Temporal lobe |
| Fibroblasts from epileptogenic focus | NCH421G | Pharmacoresistant epilepsy | Temporal lobe |
| Fibroblasts from epileptogenic focus | H158G | Pharmacoresistant epilepsy | Temporal lobe |
| CAFs from glioblastoma | NCH197G | Glioblastoma, IDHwt | Frontotemporal |
| CAFs from glioblastoma | NCH347G | Glioblastoma, IDHwt | Frontal lobe |
| CAFs from glioblastoma | NCH348G | Glioblastoma, IDHwt | Temporo parietal |
| CAFs from glioblastoma | NCH353G | Glioblastoma, IDHwt- gliosarcoma | Temporal lobe |
| CAFs from glioblastoma | NCH230G | Glioblastoma, IDHwt | Frontal lobe |
| CAFs from glioblastoma | NCH413G_M | Glioblastoma, IDHwt | Occipital lobe |
| CAFs from breast cancer brain metastasis | H117G_M | Brain metastasis of breast cancer | Cerebellum |
| CAFs from breast cancer brain metastasis | NCH233G_M | Brain metastasis of undifferentiated carcinoma, breast cancer | Frontal lobe |
| CAFs from breast cancer brain metastasis | NCH106H | Brain metastasis of breast cancer | Temporal lobe |
| CAFs from lung cancer brain metastasis | NCH402G_M | Brain metastasis of lung carcinoma | Parietal lobe |
| CAFs from lung cancer brain metastasis | NCH219G | Brain metastasis of lung adenocarcinoma | Frontal lobe |
| CAFs from lung cancer brain metastasis | H131G_M | Brain metastasis of lung adenocarcinoma | Parietal lobe |
| CAFs from lung cancer brain metastasis | NCH382G_M | Brain metastasis of small cell lung carcinoma | Frontal lobe |
| CAFs from spindle cell poorly differentiated sarcoma brain metastasis | H133G_M | Brain metastasis from spindle cell poorly differentiated sarcoma | Parietal lobe |
| CAFs from primary serous peritoneal carcinoma brain metastasis | NCH130H | Brain metastasis of high-grade primary serous peritoneal carcinoma | Occipital lobe |
| CAFs from a brain metastasis of clear cell renal carcinoma | NCH379G_M | Brain metastasis of clear cell renal carcinoma | Frontal lobe |

| **Suppl. Table 2:** Antibodies used | | |
| --- | --- | --- |
| **Antigen** | **Source** | **Identifier** |
| anti-Vimentin | Sigma-Aldrich, Prague, Czech Republic | CAT#V5255 |
| anti-Vimentin, SP20 | Abcam, Cambridge, UK | CAT#ab16700 |
| anti-Nestin, 10C2 | Abcam, Cambridge, UK | CAT#ab22035 |
| anti-TE7, TE7 | Sigma-Aldrich, Prague, Czech Republic | CAT#CBL271, |
| anti-PDGFR Beta | LS-Bio, distributed by EXBIO, Vestec, Czech Republic | CAT#11-590-C100 |
| anti-FAP, F19, mouse hybridoma | ATCC, USA | CAT#ATCC CRL-2733 (Discontinued) |
| anti-αSMA, 1A4 | Abcam, Cambridge, UK | CAT#ab7817 |
| anti-αSMA, 1A4 | Dako (Agilent), Santa Clara, CA, USA | CAT#M0851 |
| anti-CD45, HI30 | EXBIO, Vestec, Czech Republic | CAT#11-684-C100 |
| anti-GFAP, GF01 | EXBIO, Vestec, Czech Republic | CAT#11-255-C100 |
| anti-Pancytokeratin, E1/AE3+5D3 | Abcam, Cambridge, UK | CAT# ab86734 |
| anti-S100 β6, EPR13048-69 | Abcam, Cambridge, UK | CAT# ab181975 |
| anti-CD271 (anti-NGFR) | Sigma-Aldrich, Prague, Czech Republic | CAT#HPA004765 |
| anti-Ki67, MIB-1 | Dako (Agilent), Santa Clara, CA, USA | CAT# F078801 |
| anti-TBX4, PA5-65383 | Invitrogen, Waltham, Massachusetts, USA | CAT# PA5-65383 |
| anti-TBX5, PA5-29845 | Invitrogen, Waltham, Massachusetts, USA | CAT#PA5-29845 |
| anti-HOX C6, PA5-41479 | Invitrogen, Waltham, Massachusetts, USA | CAT#PA5-41479 |
| anti-HOX C8, PA5-41629 | Invitrogen, Waltham, Massachusetts, USA | CAT#PA5-41629 |
| anti-HOX D10, NBP2-45744 | Novus, Littleton, Colorado, USA | CAT#NBP2-45744 |

**Suppl. Tab. 3**: Sex effect in homeobox gene expression within selected sample groups. Only groups with at least three male and three female individuals were compared. Transcriptome profiling was performer using either RNA-seq (S) or microarray platform (A). FC – fold change, FDR – false discovery rate.

| **Sample Group** | **Platform** | **Symbol** | **Description** | **Log_2_ FC** | **FDR** | **Direction** |
| --- | --- | --- | --- | --- | --- | --- |
| **GBM** | **S** | NKX3-1 | NK3 homeobox 1 | 2.2 | 0.003 | up |
| **GBM** | **S** | HOXA10 | homeobox A10 | 4.4 | 0.005 | up |
| **GBM** | **S** | PROX1 | prospero homeobox 1 | 4.5 | 0.009 | up |
| **GBM** | **S** | EN2 | engrailed homeobox 2 | 4.6 | 0.01 | up |
| **GBM** | **S** | DLX1 | distal-less homeobox 1 | 2.6 | 0.03 | up |
| **GBM** | **S** | HOXB3 | homeobox B3 | 2.0 | 0.05 | up |
| **GBM** | **S** | HOXB7 | homeobox B7 | 1.4 | 0.06 | up |
| **GBM** | **S** | LHX2 | LIM homeobox 2 | 1.6 | 0.06 | up |
| **GBM** | **S** | HOXB4 | homeobox B4 | 1.6 | 0.07 | up |
| **GBM** | **S** | DLX2 | distal-less homeobox 2 | 1.1 | 0.08 | up |
| **GBM** | **S** | NKX2-2 | NK2 homeobox 2 | 1.1 | 0.09 | up |
| **PANF** | **A** | MEIS1 | Meis homeobox 1 | 1.1 | 0.03 | up |
| **PANF** | **A** | IRX4 | iroquois homeobox 4 | 2.7 | <0.001 | up |
| **PANF** | **A** | NKX2-5 | NK2 homeobox 5 | -2.0 | <0.001 | down |
| **PANF** | **A** | NKX3-1 | NK3 homeobox 1 | 1.4 | 0.008 | up |
| **PANF** | **A** | EMX2 | empty spiracles homeobox 2 | -1.5 | 0.005 | down |
| **DF BODY** | **A** | LHX2 | LIM homeobox 2 | 1.3 | 0.02 | up |

**
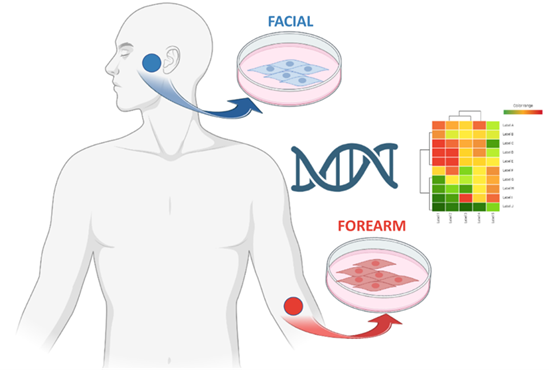
**

**Suppl. Fig. 1.** Schematic presentation of the sites from where the fibroblasts originated from the ectomesenchyme (face, 2.5 cm ventral from the tragus) and mesoderm (proximal forearm, radial side, approximately 7.5 cm distal from the flexural line) were harvested. Created with BioRender.com.

**
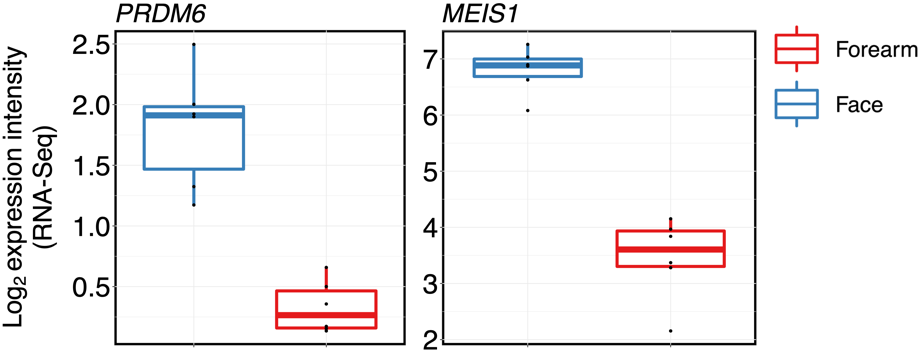
**

**Suppl. Fig. 2.**  The *PRDM6* and *MEIS1* genes are upregulated in dermal fibroblasts prepared from the face in agreement with their role in neural crest development.


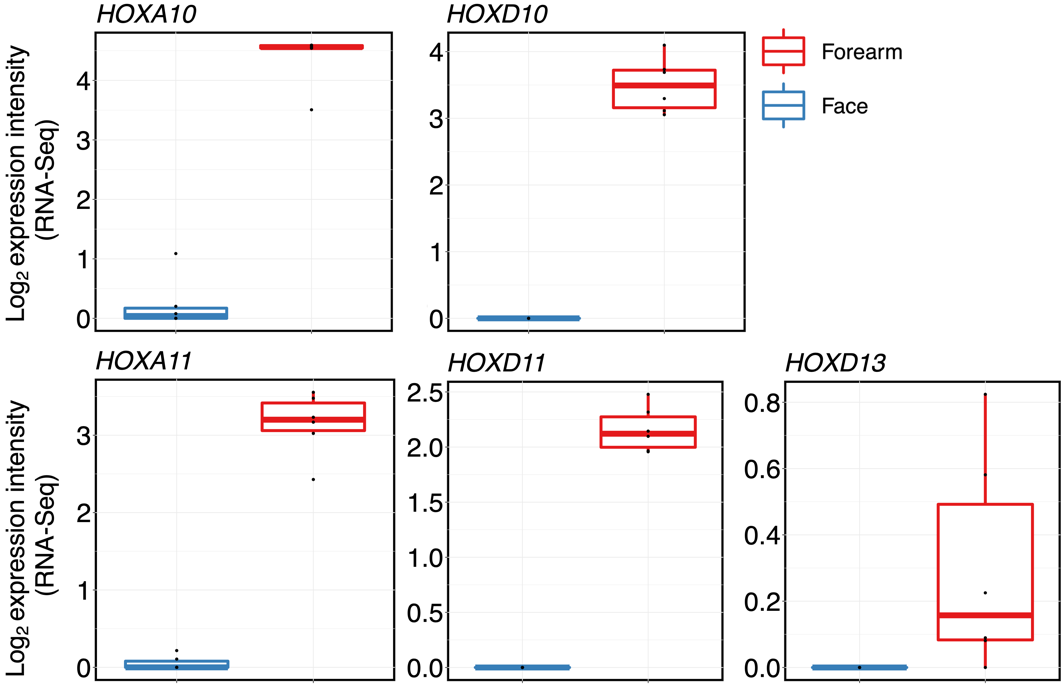


**Suppl. Fig. 3.** Upregulated genes in dermal fibroblasts prepared from the forearms that participate in the formation of the upper limb in the embryonic period.
